# Feasibility of training the dorsolateral prefrontal-striatal network by real-time fMRI neurofeedback

**DOI:** 10.1038/s41598-022-05675-0

**Published:** 2022-01-31

**Authors:** Franziska Weiss, Jingying Zhang, Acelya Aslan, Peter Kirsch, Martin Fungisai Gerchen

**Affiliations:** 1 Department of Clinical Psychology, Central Institute of Mental Health (ZI), Heidelberg University/Medical Faculty Mannheim, J5, 68159 Mannheim, Germany; 2 Department of Addiction Behavior and Addiction Medicine, Central Institute of Mental Health, Heidelberg University/Medical Faculty Mannheim, Mannheim, Germany; 3 Bernstein Center for Computational Neuroscience Heidelberg/Mannheim, Mannheim, Germany; 4 Department of Psychology, Heidelberg University, Heidelberg, Germany

**Keywords:** Psychology, Functional magnetic resonance imaging

## Abstract

Real-time fMRI neurofeedback (rt-fMRI NF) is a promising non-invasive technique that enables volitional control of usually covert brain processes. While most rt-fMRI NF studies so far have demonstrated the ability of the method to evoke changes in brain activity and improve symptoms of mental disorders, a recently evolving field is network-based functional connectivity (FC) rt-fMRI NF. However, FC rt-fMRI NF has methodological challenges such as respirational artefacts that could potentially bias the training if not controlled. In this randomized, double-blind, yoke-controlled, pre-registered FC rt-fMRI NF study with healthy participants (N = 40) studied over three training days, we tested the feasibility of an FC rt-fMRI NF approach with online global signal regression (GSR) to control for physiological artefacts for up-regulation of connectivity in the dorsolateral prefrontal-striatal network. While our pre-registered null hypothesis significance tests failed to reach criterion, we estimated the FC training effect at a medium effect size at the end of the third training day after rigorous control of physiological artefacts in the offline data. This hints at the potential of FC rt-fMRI NF for the development of innovative transdiagnostic circuit-specific interventional approaches for mental disorders and the effect should now be confirmed in a well-powered study.

## Introduction

Functional magnetic resonance imaging (fMRI) is a nowadays almost ubiquitous technique to study the brain and to gain information on alterations in brain functioning in mental disorders. With rising numbers of individuals affected by mental disorders in the recent past^1,2^ and a relevant level of non-responders to current treatments, the need not only for basic scientific results but also for novel and innovative therapy approaches based on these insights is high. Orienting towards a more treatment-related usage, fMRI has been progressively used for the application of real-time fMRI neurofeedback (rt-fMRI NF) since its’ introduction around the turn of the last century^3–5^. In rt-fMRI NF participants are trained to volitionally control a brain process, which is usually not directly accessible, in a predefined direction. The brain process is selected based on its involvement in a mental disorder and evoked changes in activity are expected to be accompanied by an improvement in symptoms^4^. rt-fMRI NF specifically profits from the high spatial resolution and whole-brain coverage^6^ provided by fMRI. Although the temporal resolution in contrast is relatively low, it still allows for feedback in nearly real-time^7,8^. As a further advantage of the method, compared to pharmacological treatments, no side effects have been reported^9^.

So far, the largest number of rt-fMRI NF studies have been conducted with Region-of-Interest (ROI) based approaches that feedback activity of a single ROI as the training target. Those studies could successfully demonstrate the feasibility of inducing changes in brain functioning (see for example^10,11^) and in several cases associated improvement in symptoms could be demonstrated^12–15^.

Notwithstanding, a transition of the focus to connectivity-based approaches has begun in the past years. First reports suggest that changes in connectivity as a consequence of rtfMRI NF can be achieved^16^ and clinical measures might be improved^17^. Recently^18^, targeted DLPFC-ACC connectivity and found increased connectivity in the experimental group which correlated with symptom improvement in high trait anxiety after rt-fMRI NF. Further technological advances are Dynamic Causal Modelling (DCM)-based NF^19,20^ and whole-brain connectome-based NF^21^.

Particularly for the development of transdiagnostic approaches addressing specific neural circuits that are involved in diverse mental disorders, like frontostriatal networks, connectivity-based rt-fMRI NF shows great promise. Before such approaches should be applied in clinical contexts, it is however necessary to address the methodological problems associated with the technology and gain a better understanding of the effects that can be expected.

In a previous single-session rt-fMRI NF study we found that FC rt-fMRI NF is heavily influenced by physiological artefacts, particularly from respiration^22^. In subsequent offline analyses of the acquired data we tested whether model-based physiology correction with the TAPAS PhysIO toolbox^23^, and global-signal regression (GSR)^24^ can eliminate the influence of these non-neural artefacts, and found that GSR is a promising approach for online physiology correction. GSR also has the further advantage that it can be implemented in a simple and straightforward manner during the estimation of the online feedback signal.

In the present double-blind randomized yoke-controlled pre-registered study we now tested in healthy controls over three training days whether an updated rt-fMRI NF approach with online GSR can be used to train participants to up-regulate FC in a bilateral frontostriatal network comprising the DLPFC and the striatum. With this study we aimed at demonstrating the principle feasibility of this approach, gain insight into the time course of the training effect, obtain basic effect size estimates and by this pave the way for future confirmatory studies and clinical applications of the developed technology to modulate frontostriatal circuitry in the diverse clinical conditions where they are involved.

## Methods

### Participants

40 healthy participants took part in this double-blind randomized yoke-controlled rt-fMRI NF experiment. Participants (24 female, 16 male) were between 19 and 30 years of age (mean: 23.28; SD: 2.39), did not present with any current or prior psychiatric diagnosis, had normal or corrected-to-normal vision, were free of a history of mental and neurological disorders and were not on acute psychopharmacological medication. Female participants were not pregnant. The study was approved by the Ethics Committee of the Medical Faculty Mannheim at the University of Heidelberg, Germany (2018-520N-MA) and complies with the World Medical Association’s Declaration of Helsinki. Informed consent was obtained from all participants.

### Pre-registration

The study was pre-registered at the Open Science Foundation (OSF NeCoSchi II https://osf.io/znrbk/). Specifically, we tested here the following two pre-registered hypotheses:

1. Averaged correlations between DLPFC and striatum are higher in the real neurofeedback group in comparison to the yoke control group during rt-fMRI neurofeedback sessions (directional).
2. Participants from the real neurofeedback group in contrast to participants from the yoke control group will demonstrate a higher increase of averaged correlations between DLPFC and striatum from the initial resting state period (directional).

### MRI scanning

MRI scanning was administered at a Siemens Biograph Scanner with 3 T (Siemens Healthineers, Erlangen, Germany) at the Central Institute of Mental Health in Mannheim, Germany. MR images were acquired with a 32-channel head coil. T1-weighted structural images were obtained with a repetition time (TR) of 2 s, echo time (TE) = 2.58 ms, flip angle = 10°, 192 slices, slice thickness = 0.9 mm, voxel dimensions = 0.4 mm × 0.4 mm × 0.9 mm, FoV = 192 mm. Echo planar imaging (EPI) sequences were acquired with a TR of 1.64 s, TE = 30 ms, flip angle = 73°, 30 slices, slice thickness = 3 mm, voxel dimensions = 3 mm × 3 mm × 3 mm, FoV = 192 mm, GRAPPA factor 2. 343 Volumes were acquired and EPI sequence was the same for all functional runs. Physiological signals were measured with built-in equipment during functional scans.

### Brain network definition

**Figure 1:**
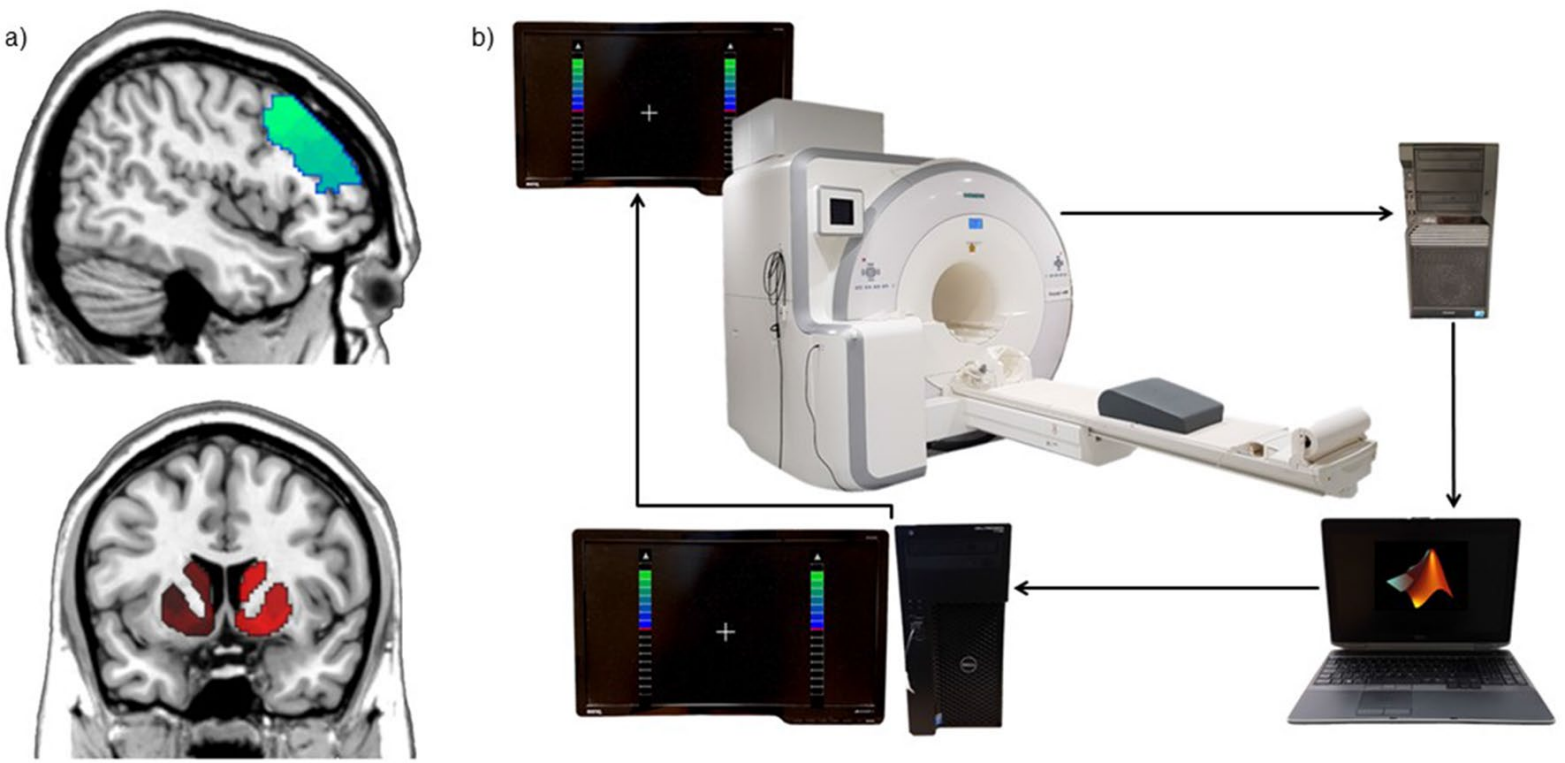
Experimental setup. (**a**) DLPFC-striatum target network. Bilateral ROIs in the DLPFC and the striatum were predefined and projected into the individual anatomy of the participant to extract the online feedback signal during NF training. (**b**) rt-fMRI NF setup. Images are sent to a laptop running in-house MATLAB scripts for pre-processing and extraction of the neurofeedback signal. The feedback signal represents the averaged functional connectivity between the ROIs in the DLPFC and the striatum. The feedback value is forwarded to a computer running Presentation software and is displayed in the scanner as a thermometer value that is continuously updated every TR.

The rt-fMRI NF approach and all analyses focused on a predefined bilateral network including the dorsolateral prefrontal cortex (DLPFC) and the striatum (see Fig. 1a). 22 ROIs in the DLPFC and 12 ROIs in the striatum were extracted from the cortical parcellations by^25^ and the striatal parcellation by^26^ which are both based on the 7-network cortex parcellation by^27^. The DLPFC ROIs were identified based on an automatic metaanalysis with Neurosynth (https://neurosynth.org/^28^; with the term “DLPFC”. We selected a broad measure of DLPFC-striatum FC as feedback target instead of targeting specific striatal subnetworks. Cortical regions have widespread projection fields in the striatum which enables them to influence other networks^29^), and the location of maximal connectivity is dynamically changing over the striatum^30^). Thus, with our approach we are aiming at training the general ability of the DLPFC to exert control over striatal processes without focusing on a specific sub-network. However, our approach based on several ROIs within the target regions would allow for the identification of potential sub-networks linked with symptom changes in clinical studies. This could then facilitate refinement of the target networks. It is further important to note that while we used averaged connectivity as a rather simple network measure in this study our approach provides the technological basis for future NF applications that could take more complex graph-theoretical network measures into account.

### rt-fMRI NF training

For a graphic representation of the NF-setup, please refer to Fig. 1b. NF training was performed on three separate fMRI scanning days. At the start of the first day, demographic information was collected and questionnaires were answered. The questionnaires included the German Version of the Beck Depression Inventory (BDI-II)^31^, the Schizotypal Personality Questionnaire (SPQ)^32^, the 10 Item Big Five Inventory (BFI-10)^33^ and a sensory inventory^34^. The first and the third scanning day comprised a T1-weighted anatomical MPRAGE scan (5 min), an initial resting state scan, two NF runs and a transfer run with each one being of 9:29 min length. On the second training day the third run was also a NF run instead of a transfer run. The initial resting state run was conducted with open eyes while a fixation cross was displayed at the center of the screen. The transfer run aims at testing for the generalizability of NF learning without a feedback signal and was in essence equal to the resting state run with the difference that participants were instructed to regulate their brain as in the NF run. The NF run included the presentation of a fixation cross which was located in the center of the screen. The fixation cross was surrounded by two thermometer bars that indicated the value to be up-regulated. The value was updated every TR and consisted of the averaged Z-transformation Pearson’s correlation of DLPFC and striatal ROIs. Participants were not informed about specific mental strategies but were instructed to try out different strategies and were told to pursue the one they find most successful. Participants were randomly assigned to the real (N = 20) or yoke control group (N = 20) in a double-blind fashion by an automatic procedure implemented in the MATLAB code with a pre-specified randomization list. Participants in the yoke control group were paired with unique participant from the real group with a first-in-first-out procedure and received in each run the saved feedback signal of this participant from the same run. Data processing and handling were the same for both groups; just the sent feedback signal was automatically replaced in the yoke group. Thus, the staff was unaware of the group identity of the participants. The received feedback signal was consistent between the real and yoke control group in every run. The first three participants were deliberately allocated to the real group to ensure that sufficient recordings for the yoke procedure were available. After each scanning session participants rated their subjective performance, reported used strategies, and indicated which group they thought they belong to. At the end of the last session group allocation was disclosed.

### Online data analysis

Online and offline analyses were conducted in MATLAB (R2019a, Math Works Inc., Sherborn, MA, USA). In-house software based on SPM functions^22,35^ was used for online rt-fMRI NF processing. During the resting state scan the anatomical image was segmented and normalized to the SPM 12 TPM MNI template. ROI masks were then projected into individual subject space. For rt-fMRI processing, during scanning every collected volume was immediately transferred to the laptop where the analysis was run. Each volume was realigned to the first volume of the series and averaged intensity values from all ROIs were extracted and added to the ROI signal time series. A general linear model (GLM) was estimated at every step over all acquired data to correct for movement parameters, a cerebrospinal fluid (CSF) signal, spikes correlated with head movements (framewise displacement (FD) > 0.5 mm) and the global signal. The last 15 volumes were used to calculate Fisher Z-transformed Pearson correlations (i.e. achieving a sliding window size of 15 volumes) between all ROIs in the DLPFC and all ROIs in the striatum. These were then averaged to obtain the online feedback signal. The first feedback value was presented after ~ 1 min (37 volumes = 60.68 s). This delay was included to ensure that sufficient data for a stable estimation of the nuisance regression model was available. Only windows including a minimum of 10 volumes that were not influenced by head motions (FD < 0.5 mm) were used for the calculation of the feedback signal. If a window contained insufficient information the feedback value was kept constant.

### Offline data analysis

SPM 12 (v6906) was used for offline data analysis. The anatomical image was segmented and normalized to SPM 12 TPM MNI space. We removed the first 10 volumes of the functional data. The images were slice-time corrected, realigned to the mean image, and co-registered to the anatomical image. The images were normalized, scaled to a resolution of 2 × 2 × 2 mm and smoothed with an isotropic Gaussian kernel of 6 mm full width at half maximum. In a first level General Linear Model (GLM) six movement parameters, the cerebrospinal fluid (CSF) signal, dummy regressors for volumes affected by head motion identified with the ART toolbox (framewise displacement > 0.5 mm; scan-to-scan global signal change z > 4, physiological nuisance regressors (see next paragraph)) and a constant were included. Runs with > 20% movement-affected volumes were excluded from further analyses.

### Physiological noise correction

A built-in respiration belt and a pulse finger clip (PMU Wireless Physio Control, Siemens Healthineers, Erlangen, Germany) were utilized for recording of respiration and heart rate during MRI scanning with a sampling rate of 400 Hz. To allow for evaluation of physiological parameters, physiological recordings were cut on the basis of recorded volume triggers for precise alignment with the fMRI data. Next, the TAPAS PhysIO Toolbox^23^ was applied for estimation of 20 physiological nuisance regressors, including heart rate variability (HRV), respiratory volume per time (RVT) and cardiac × respiratory interaction. Deduced physiology nuisance regressors were implicated in the first level GLM of the analyses.

### Respiratory parameters

To assure that our results were not confounded by respiratory artifacts^22^ and demonstrate that GSR and model-based physiology correction worked efficiently in cleaning up the data, we further computed summarizing respiratory parameters from the time courses that are possibly related to the BOLD signal^22,36^. Breath Rate that is defined as peaks/breaths per minute and Pause CV which is the coefficient of variance of respiration pause duration were calculated. For a more detailed description, see^22,36^.

### Offline connectivity estimates

Offline connectivity was estimated over the same averaged ROI-to-ROI connections as in the online approach, but over the whole available time course. For testing the second hypothesis the connectivity estimates of the initial resting state period of each day were subtracted from the respective estimates of the NF and transfer runs within each participant to normalize the modulation effect with respect to the individual baseline.

### Second level analyses

Second level analyses were performed based on the DLPFC-striatal large-scale network connectivity values. For each NF and transfer run, connectivity estimates corrected for age and gender as covariates were compared between the two groups with one-sided independent samples t-tests implemented in a GLM model. Hedges’g and its confidence interval were estimated based on the obtained t-values to estimate the effect size per run^37^. Pearson’s correlations were used to assess associations of offline and online connectivity with respiratory parameters.

## Results

### Functional connectivity group comparison

The randomized groups did not differ in terms of age (t(38) = 0.1964, p = 0.8454) and gender, x^2^(1, N = 40) = 1.667, p = 0.197). Participants were not able to indicate above chance which group they were assigned to (training day1: x^2^(1, N = 39) = 0.205, p = 0.651; training day2: x^2^(1, N = 40) = 0.404, p = 0.525; training day3: x^2^(1, N = 40) = 1.616, p = 0.204).

**Figure 2:**
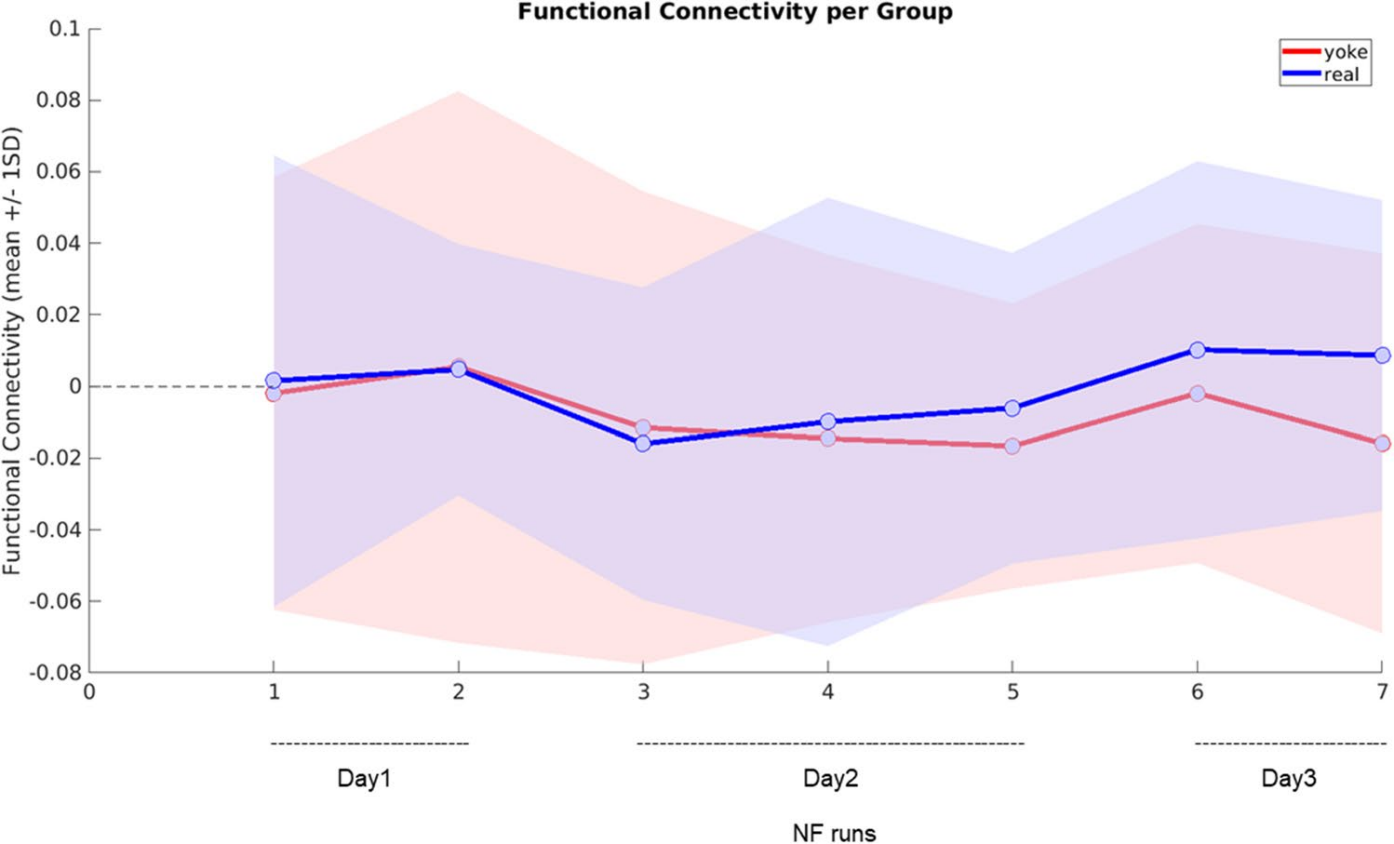
Functional connectivity over runs. Functional connectivity per group during NF runs normalized by initial resting state FC of the respective day and corrected for age and gender is displayed (blue = real feedback group, red = yoke feedback group). Shaded areas represent ± 1SD from the group mean. 7 NF training runs were conducted over three training days. A moderate group difference was found during NF run 7 (the last NF run of day3).

**Figure 3:**
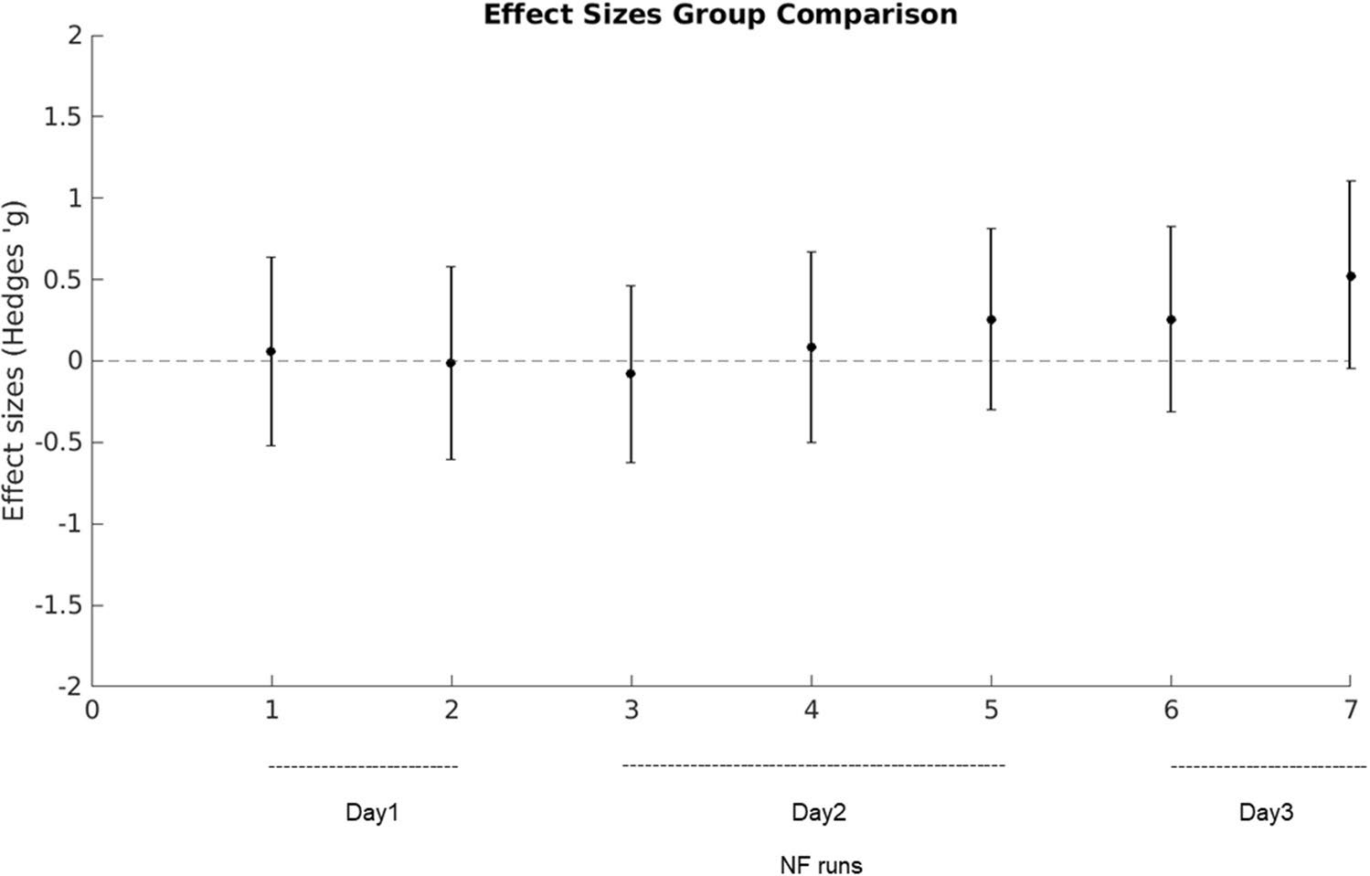
Effect sizes of the group comparison. Effect sizes (Hedges’ g with 90% confidence interval) of the group comparison testing for differences in DLPFC-striatum FC between the real feedback group and the yoke control group for each NF run. An effect of g = 0.5206 was found in NF run 7 at day 3.

In line with our first pre-registered hypothesis, we investigated whether absolute averaged correlations between DLPFC and striatum were increased in the real group in comparison to the yoke control group. Here, no significant group differences could be found (see Supplementary Table 1). In accordance with our second pre-registered hypothesis we further investigated whether the real group would present with an increased relative FC in the NF runs normalized to individual baseline FC in the initial resting state run of the respective day. On a purely descriptive level, while at the start of the training FC in the target network was similar in the two groups, this began to change on the second training day (Fig. 2). From the second NF run on day 2 on, the real group shows higher connectivity than the yoke group and this difference augments until the end of the training. We also did not find any significant group differences with the pre-registered null hypothesis significance tests at the specified criterion of p < 0.05. In the second NF run on the third scanning day, which was the last of all conducted NF runs, significance testing led to a result of t(33) = 1.5469, p = 0.0657 (see Table 1) for an effect with a medium effect size (Hedges’ g = 0.5206) that however had an accordingly large 90% confidence interval ranging from a very small to a medium effect (Fig. 3).

**Table 1:** Group comparisons of DLPFC-striatum functional connectivity per run corrected for age and gender as covariates.

Runs	Group differences functional connectivity
Day1_NF1	t(31) = 0.1685, p = 0.43365
Day1_NF2	t(30) = − 0.0370, p = 0.48535
Day1_transfer	t(31) = − 0.5548, p = 0.2915
Day2_NF1	t(35) = − 0.2495, p = 0.4022
Day2_NF2	t(31) = 0.2456, p = 0.4038
Day2_NF3	t(33) = 0.7689, p = 0.2237
Day3_NF1	t(33) = 0.7515, p = 0.2288
Day3_NF2	t(33) = 1.5469, p = 0.0657
Day3_transfer	t(32) = 1.2275, p = 0.1143

Functional connectivity was normalized by initial resting state activity of the respective day.

The effect in the subsequent transfer run was also not significantly different in the two groups, (t(32) = 1.2275, p = 0.1143) and had a slightly smaller effect size of Hedges’ g = 0.4199.

### Physiological associations

We further explored the presence of remaining physiological associations after the online and offline processing procedures. In the offline data, over all NF and transfer runs, we identified one run in the real group in which FC was associated with respiration and one run in which the same applied to the yoke group (Pause CV for both) (see Supplementary Table 2). Unfortunately, more physiological associations were present in the online data. Within the real group we found four significant associations (Pause CV: 2, Breath Rate: 2) and the same was true for the yoke group. For further details, please refer to the supplement (Supplementary Table 3). It is important to note here that our effect size estimates are based on the offline data, and that no physiological associations were present in run 2 of day 3 in which we detected moderate evidence against the null (offline association over whole sample: Breath rate: rho = 0.043, p = 0.803; Pause CV: rho = − 0.0182, p = 0.287, see Supplementary Fig. 3 for more details).

## Discussion

In this study we tested the feasibility of functional connectivity NF training in a large-scale DLPFC-striatal network and estimated a medium effect size for the difference between the experimental (real) and control (yoke) group at the end of the third training day. Throughout the study we aimed at rigorously controlled experimental procedures including pre-registration, online physiology correction, double-blind randomization, and a yoke control group.

Unfortunately, our pre-registered null hypothesis significance tests failed to reach significance at the specified criterion of p < 0.05, and thus do not provide clear evidence against the null hypothesis. This is likely due to our study being clearly underpowered. When we planned the study we were not having a good estimate of the expected effect size and thus planned the study with the minimum reasonable sample size with the intention to obtain effect size estimates from the study. It is important to note that it is still rather common for current NF studies to be underpowered^6^. While our sample size of N = 40 in two groups is small, it is not one of the smallest in the field of rt-fMRI NF (see for example^4,38,39^). To address this issue in future research it is important to have realistic estimates of effect sizes in rt-fMRI NF^40^. Our medium effect size of Hedges’ g = 0.5206 is similar to the estimation of^41^ who found medium effect sizes (Hedges’ g = 0.59) for regulation over all included NF studies and^6^ who showed that an effect size of 0.73 (Cohen’s d) can be detected with a power of 95% based on ROI activation and connectivity regulation NF studies.

The time course of the NF training effect in our data looks promising. Increased FC in the experimental group in contrast to the yoke group is on a purely descriptive level visible from the second NF training run of day 2 and then increases until the end of the third day.

The trend of this time course suggests that the effect might even increase when additional training days would be added. Our findings fit into the framework of a growing number of methodological papers that investigate optimal conditions for rt-fMRI NF^42–44^ and are in line with^18^ and^45^ which demonstrate that NF training effects begin to appear after two training days. Along these lines, in an extensive NF study including 12 training days^46^, found an increase in training efficiency as far as the 3rd–4th day. Thus, single-session NF training might not be sufficient, although some studies find effects in just one training session^9,47,48^.

On these grounds, our effect size estimated prepare the ground for an adequately powered confirmatory study to validate the time course and the effect size of the training effect, which should include at least one further training day. A reason for the non-significant results despite a medium effect size and a decent sample size might be a high signal-to-noise ratio in the data. One potential source of interindividual noise might be the use of a yoke control procedure together with a target process involved in executive control. It could be that perceived group assignment or the dynamics of the feedback signal might have had an influence on the target process in our study, especially in the yoke control group.

Importantly, when comparing NF studies, a differentiation should be made between activation NF and FC NF as the latter is a more complex signal and this probably harder to train.

(Online) control of confounding noise sources is essential for FC rt-fMRI NF^22,43^. Importantly, unintended sources should optimally be corrected in the data already in the online analyses, because of their potential to bias the training if the feedback value is confounded. In our previous study we identified GSR as a promising approach to clean up the online signal^22^, and implemented it in the present experiment. GSR is however a controversial method^49,50^ and has been criticized for being too rigid potentially removing real signal along with noise^51^ and might thus reduce the power of the study, i.e. the probability to uncover real NF effects. On the other side, GSR is one of the most efficient methods for correction of global artefacts and has been recommended for correction of respiratory noise, for example in modern multiband sequences^52,53^.

Unfortunately, despite the application of online GSR, in some runs associations of the online signal with respiratory measures were still present in the data (see Supplementary Table 3). For additional investigation of physiology correction and a comparison between artefactual physiological artefacts offline and online, we conducted analyses without specific physiology correction (without GSR and Physio) and GSR only (without PhysIO) (see Supplementary Tables 4 and 5). These findings replicate the findings in the preceding paper^22^. Most importantly, the offline analyses without GSR and PhysIO correction identified substantial physiological associations in the vast majority of runs (21/28 “corrupt” runs), which is much higher than the number of associations in the online training data. This clearly demonstrates that our online correction machinery with GSR was more effective in cleaning the data than the usual offline analysis without further correction. GSR thus seems to have a strong incremental value despite its controversial aspects and can be recommended for online use, although it is slightly less effective online. Regarding more insights into the relationship of the different processing strategies, an exemplary network connectivity time course with the different processing strategies is shown (see Supplementary Fig. 4). This figure demonstrates the strong influence of GSR on the network connectivity estimate and why it has such a strong influence on the detection of physiological associations. While GSR might be a helpful tool in addressing physiological artefacts, it does not solve the issue completely and further improvements are necessary, for example by online implementation of model-based physiology correction algorithms using simultaneously acquired physiological signals^54^. Such approaches are however technically much more demanding than simple GSR and are not available yet.

It is important to note that in the offline analyses we additionally conducted model-based physiology correction with the TAPAS PhysIO toolbox^23^ before we calculated FC estimates. After this additional correction, associations with respiratory measures were widely diminished (Supplementary Table 2), but the reported evidence of a NF training effect was still present. Thus, GSR alone might not already be the optimal method to control online physiological artefacts, but our NF approach seems at least sufficient for generating evidence of FC training effects beyond physiological artefacts.

A further methodological rigor of our experiment is the use of a double-blind yoke-controlled design. A majority of rt-fMRI NF studies is conducted single-blind (see for example^13,55,56^) and double-blind designs are still rare, although they are highly recommended, for example in the CRED-nf protocol for neurofeedback studies^57^.

While the recommendation for a double-blind procedure is unambiguous, several different control procedures for NF studies are available. It is for example possible to use computer-generated sham feedback in the control group^58^ or employ a within-subjects design in which participants received real feedback in the first session and control feedback in the second session^17^. Our selected yoke-control procedure guaranteed that all facets of the conditions apart from control over the ROI signal were matched^44^. An important aspect of the interpretation of results from a yoke-controlled design is whether participants can accurately indicate which group they were assigned to. If participants in the yoke control group are able to correctly identify themselves, this could cause frustration and influence performance. Accordingly, this could artificially inflate group differences. However, as our participants were not able to accurately guess their group identity, this was likely not a problem in our study.

We used a design with continuous NF regulation over whole runs because this corresponded well with the requirements of the FC-based feedback measure. However, the task was demanding and fatigue might have prevented a better performance. It remains open whether a block design with alternating NF and rest blocks would have provided a better training outcome.

We estimated FC based on a sliding window of 15 volumes (24.6 s). This is a window size within the normal range in the field of FC NF but can introduce a substantial delay in the feedback signal. We chose this window size because it covers the full waveform of the canonical hemodynamic response function, leaves sufficient data for estimating connectivity even when several volumes are censored, and corresponds to the continuous nature of our training. It can however not be excluded that a shorter window might have provided better learning and future research is needed to empirically address the influence of the window size on NF learning.

Of note, the FC values were normalized by the individual baseline FC in the initial resting state scan of the respective day (hypothesis two). Instead of taking the resting state scan as the baseline it would be another possibility to conduct the transfer run before the NF runs and use this pre-training transfer as baseline. We conclude that it is important and particularly more sensitive to take an individual baseline into account when calculating group differences in FC.

In comparison to the last NF run on training day three, the effect size was smaller in the transfer run without a feedback signal (Supplementary Figs. 1 and 2) again suggesting that additional training runs might improve the effects. Nonetheless, our findings might cautiously be interpreted in a way that successful regulation in the transfer task could potentially be achieved after further training.

The patterns in our data, if confirmed, would underline the potential of FC rt-fMRI NF to induce actual changes in FC beyond physiological artefacts and thus provide various options to develop innovative and transdiagnostic treatment approaches for different mental disorders sharing common neural features like frontostriatal dysconnectivity. However, it is still to demonstrate that changes in FC also lead to changes in behavioral or disorder associated alterations. Given the relatively high scanning costs, rtfMRI NF is however a rather expensive method. On the other hand, rt-fMRI NF is completely non-invasive, is able to address very specific and complex phenotypes in the brain, and has the potential to evoke changes in FC^59^. To avoid unnecessary costs, it will be important to identify predictors of successful NF training (see for example^14,60^) to inform precise individualized treatment approaches.

## Conclusion

Our findings extend the hitherto thin but increasing literature on connectivity fMRI NF studies^9,18,61,62^ by adding effect size estimates for NF modulations of complex fMRI signals. The moderate effects could only be seen after extensive training and several FC rt-fMRI NF training sessions. Extra caution is needed in controlling the online target signal for artefacts. Overall, our study supports the further exploration of FC-based rt-fMRI NF as a contingently promising method to develop circuit-specific treatment approaches for mental disorders in adequately powered confirmatory studies.

## Supplementary Information

Supplementary Information.

## Data Availability

The data that support the findings of this study are available in the Open Science Foundation (OSF) repository, at: https://osf.io/znrbk/. Supplementary data to this article can be found online at 10.1038/s41598-022-05675-0.
