# Supplementary material for: Feasibility of training the dorsolateral prefrontal-striatal network by real-time fMRI neurofeedback
Source: Sci Rep. 2022 Jan 31;12:1669. doi: 10.1038/s41598-022-05675-0 (PMC8803939; doi:10.1038/s41598-022-05675-0)
Supplement: Supplementary file 1 — Supplementary Information. [file 41598_2022_5675_MOESM1_ESM.docx]

**Supplement**

Feasibility of training the dorsolateral prefrontal-striatal network by real-time fMRI neurofeedback

Franziska Weiss, Jingying Zhang, Acelya Aslan, Peter Kirsch, Martin Fungisai Gerchen

**Supplementary Table 1.** Group comparisons of functional connectivity per run corrected for age and gender as covariates. Functional connectivity was not corrected for baseline resting state activity.

| **Runs** | **Group Differences Functional Connectivity** |
| --- | --- |
| Day1_NF1 | t(32) = -0.0082  p = 0.49675 |
| Day1_NF2 | t(30) = -0.2884  p = 0.38755 |
| Day1_transfer | t(31) = -0.2995  p = 0.3833 |
| Day2_NF1 | t(36) = -0.5687  p = 0.28655 |
| Day2_NF2 | t(31) = -0.4512  p = 0.3275 |
| Day2_NF3 | t(33) = -0.2920  p = 0.38605 |
| Day3_NF1 | t(35) = 0.4108  p = 0.34185 |
| Day3_NF2 | t(33) = 1.2299  p = 0.1137 |
| Day3_transfer | t(33) = 0.8042  p = 0.2135 |

|  | **Real** | | **Yoke** | |
| --- | --- | --- | --- | --- |
|  | **Respiratory measures** | | **Respiratory measures** | |
| **Runs** | **Pause CV** | **Breath Rate** | **Pause CV** | **Breath Rate** |
| Day1_NF1 | rho = -0.1885  p = 0.4537 | rho = -0.2536  p = 0.3100 | rho = 0.2253  p = 0.3847 | rho = 0.0578  p = 0.8256 |
| Day1_NF2 | rho = -0.4650  p = 0.0939 | rho = -0.3912  p = 0.1666 | rho = -0.2721  p = 0.2597 | rho = 0.1207  p = 0.6226 |
| Day1_transfer | rho = -0.5911  p = 0.0203* | rho = -0.0976  p = 0.7293 | rho = -0.1508  p = 0.5503 | rho = 0.4324  p = 0.0731 |
| Day2_NF1 | rho = -0.0280  p = 0.9093 | rho = -0.1887  p = 0.4839 | rho = 0.3652  p = 0.1241 | rho = -0.3439  p = 0.1494 |
| Day2_NF2 | rho = -0.2716  p = 0.3089 | rho = -0.0239  p = 0.9301 | rho = 0.6572  p = 0.0030* | rho = -0.0879  p = 0.7288 |
| Day2_NF3 | rho = -0.2254  p = 0.4013 | rho = -0.3271  p = 0.2162 | rho = -0.3212  p = 0.1799 | rho = 0.2630  p = 0.2767 |
| Day3_NF1 | rho = -0.4378  p = 0.0608 | rho = 0.3303  p = 0.1673 | rho = -0.1536  p = 0.5301 | rho = 0.1542  p = 0.5285 |
| Day3_NF2 | rho = -0.2707  p = 0.2933 | rho = -0.1840  p = 0.4797 | rho = -0.0182  p = 0.9412 | rho = 0.2095  p = 0.3893 |
| Day3_transfer | rho = -0.3791  p = 0.1475 | rho = 0.2949  p = 0.2675 | rho = -0.1079  p = 0.6701 | rho = 0.3807  p = 0.1191 |

**Supplementary Table 2.** Associations of offline functional connectivity with respiratory measures displayed per group.

**Supplementary Table 3.** Associations of online functional connectivity with respiratory measures displayed per group.

|  | **Real** | | **Yoke** | |
| --- | --- | --- | --- | --- |
|  | **Respiratory measures** | | **Respiratory measures** | |
| **Runs** | **Pause CV** | **Breath Rate** | **Pause CV** | **Breath Rate** |
| Day1_NF1 | rho = 0.6498  p = 0.0035* | rho = 0.1172  p = 0.6432 | rho = 0.4511  p = 0.0692 | rho = -0.3838  p = 0.1283 |
| Day1_NF2 | rho = -0.1008  p = 0.7317 | rho = -0.2795  p = 0.3332 | rho = -0.1328  p = 0.5877 | rho = -0.2012  p = 0.4089 |
| Day1_transfer | - | - | - | - |
| Day2_NF1 | rho = -0.0359  p = 0.8840 | rho = -0.3755  p = 0.1132 | rho = 0.4821  p = 0.0366* | rho = -0.5795  p = 0.0093* |
| Day2_NF2 | rho = 0.0558  p = 0.8373 | rho = -0.3524  p = 0.1806 | rho = 0.3124  p = 0.2069 | rho = -0.3479  p = 0.1571 |
| Day2_NF3 | rho = 0.3974  p = 0.1275 | rho = -0.5630  p = 0.0232* | rho = -0.2574  p = 0.3024 | rho = -0.3408  p = 0.1664 |
| Day3_NF1 | rho = 0.1403  p = 0.5669 | rho = -0.3422  p = 0.1515 | rho = 0.4510  p = 0.0526 | rho = -0.4309  p = 0.0655 |
| Day3_NF2 | rho = 0.7444  p = 6.1052e-04* | rho = -0.7031  p = 0.0016* | rho = 0.5000  p = 0.0293* | rho = -0.6471  p = 0.0027* |
| Day3_transfer | - | - | - | - |

|  | **Real** | | **Yoke** | |
| --- | --- | --- | --- | --- |
|  | **Respiratory measures** | | **Respiratory measures** | |
| **Runs** | **Pause CV** | **Breath Rate** | **Pause CV** | **Breath Rate** |
| Day1_NF1 | rho = 0.7636  p = 2.2615e-04* | rho = -0.0746  p = 0.7686 | rho = 0.6176  p = 0.0082* | rho = -0.5231  p = 0.0312* |
| Day1_NF2 | rho = 0.7277  p = 0.0032* | rho = 0.1915  p = 0.5120 | rho = 0.4607  p = 0.0472* | rho = -0.6268  p = 0.0041* |
| Day1_transfer | rho = 0.4715  p = 0.0761 | rho = -0.4244  p = 0.1148 | rho = 0.6327  p = 0.0036* | rho = -0.4354  p = 0.0624 |
| Day2_NF1 | rho = 0.7473  p = 2.3544e-04* | rho = -0.2061  p = 0.3974 | rho = 0.8777  p = 7.9500e-07* | rho = -0.8073  p = 2.9321e-05* |
| Day2_NF2 | rho = 0.5822  p = 0.0142* | rho = -0.1632  p = 0.5315 | rho = 0.7990  p = 4.0792e-05* | rho = -0.7416  p = 2.7910e-04* |
| Day2_NF3 | rho = 0.7840  p = 3.2488e-04* | rho = -0.1934  p = 0.4729 | rho = 0.0282  p = 0.9088 | rho = -0.6168  p = 0.0049* |
| Day3_NF1 | rho = 0.5763  p = 0.0098* | rho = -0.5674  p = 0.0113* | rho = 0.6262  p = 0.0041* | rho = -0.5786  p = 0.0095* |
| Day3_NF2 | rho = 0.8355  p = 2.9782e-05* | rho = -0.4874  p = 0.0472* | rho = 0.4082  p = 0.0827 | rho = -0.6110  p = 0.0055* |
| Day3_transfer | rho = 0.7328  p = 0.0012* | rho = -0.4997  p = 0.0487* | rho = 0.4096  p = 0.0816 | rho = -0.6258  p = 0.0042* |

**Supplementary Table 4.** Associations of offline functional connectivity with standard pre-processing with respiratory measures displayed per group.

|  | **Real** | | **Yoke** | |
| --- | --- | --- | --- | --- |
|  | **Respiratory measures** | | **Respiratory measures** | |
| **Runs** | **Pause CV** | **Breath Rate** | **Pause CV** | **Breath Rate** |
| Day1_NF1 | rho = -0.1348  p = 0.5940 | rho = -0.3541  p = 0.1494 | rho = 0.1376  p = 0.5986 | rho = 0.1571  p = 0.5470 |
| Day1_NF2 | rho = -0.4013  p = 0.1549 | rho = -0.4605  p = 0.0975 | rho = -0.3021  p = 0.2087 | rho = 0.1660  p = 0.4971 |
| Day1_transfer | rho = -0.5911  p = 0.0203* | rho = -0.1170  p = 0.6779 | rho = -0.2034  p = 0.4035 | rho = 0.4568  p = 0.0493* |
| Day2_NF1 | rho = -0.0163  p = 0.9473 | rho = -0.2119  p = 0.3838 | rho = 0.4550  p = 0.0503 | rho = -0.3952  p = 0.0940 |
| Day2_NF2 | rho = -0.3889  p = 0.1229 | rho = 0.0316  p = 0.9042 | rho = 0.5811  p = 0.0091* | rho = -0.0468  p = 0.8491 |
| Day2_NF3 | rho = -0.1128  p = 0.6775 | rho = -0.3295  p = 0.2126 | rho = -0.3035  p = 0.2066 | rho = 0.2410  p = 0.3202 |
| Day3_NF1 | rho = -0.4167  p = 0.0760 | rho = 0.3425  p = 0.1512 | rho = -0.1684  p = 0.4908 | rho = 0.1130  p = 0.6450 |
| Day3_NF2 | rho = -0.2151  p = 0.4069 | rho = -0.3508  p = 0.1674 | rho = -0.0335  p = 0.8917 | rho = 0.2514  p = 0.2992 |
| Day3_transfer | rho = -0.3326  p = 0.2082 | rho = 0.0356  p = 0.8959 | rho = -0.0696  p = 0.7771 | rho = 0.3701  p = 0.1188 |

**Supplementary Table 5.** Associations of offline functional connectivity with GSR only with respiratory measures displayed per group.


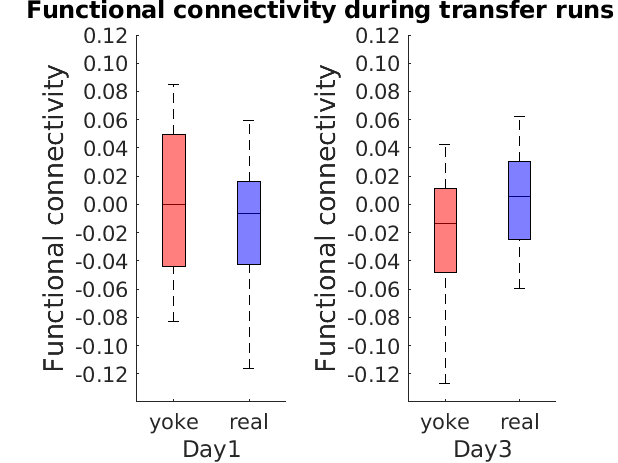


**Supplementary Figure 1.** Group comparisons of Functional Connectivity (FC) during transfer. Transfer runs were conducted on the first (t(31) = -0.5548, p = 0.2915) and third training day (t(32) = 1.2275, p = 0.1143). FC is normalized by initial resting state FC of the respective day and corrected for age and gender.


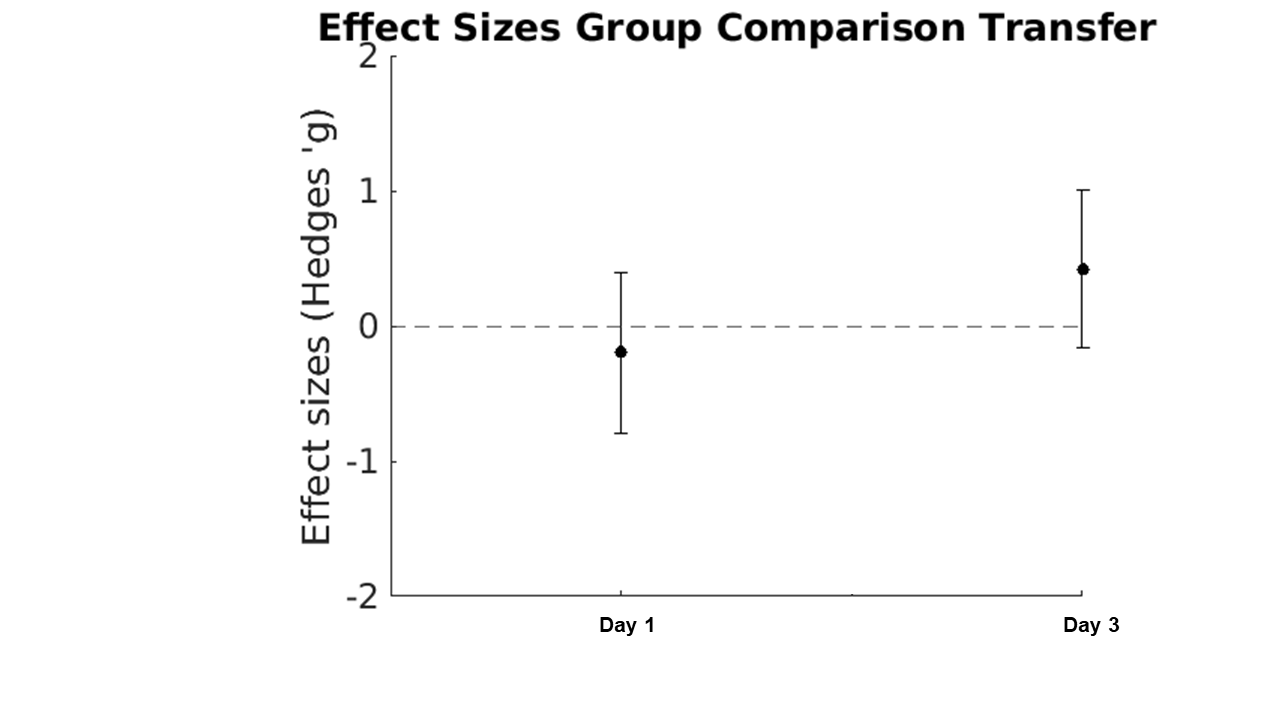
**Supplementary Figure 2.** Effect sizes of the group comparisons during transfer runs. Transfer runs were conducted on the first (g = -0.1960) and third training day (g = 0.4199).


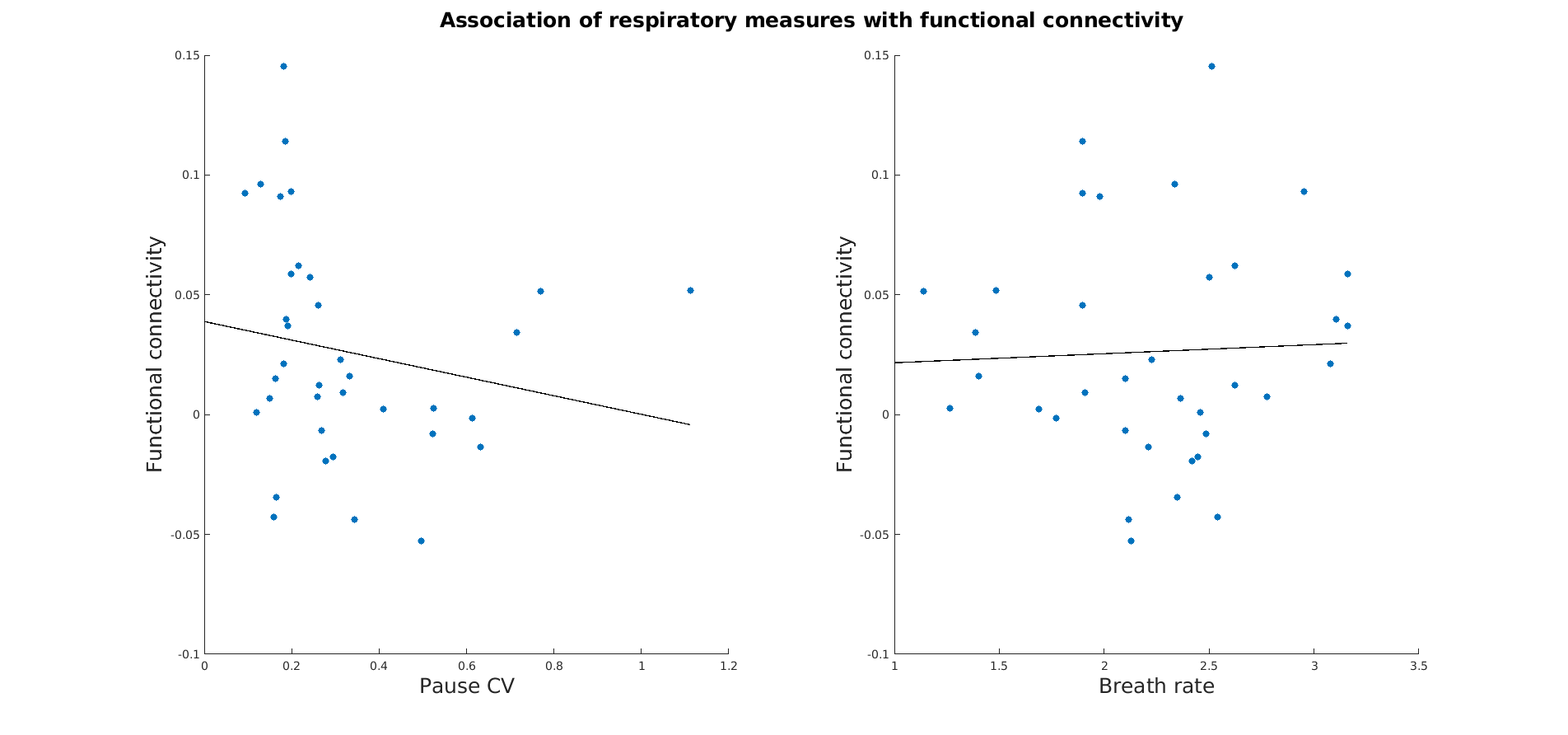


**Supplementary Figure 3.** Relationship of respiratory measures with functional connectivity (FC) in the second NF run of the third training day. The left coordinate system displays FC in relation to Pause CV (rho = -.0182, p = 0.287). On the right, the association with Breath rate is shown (rho = 0.043, p = 0.803).


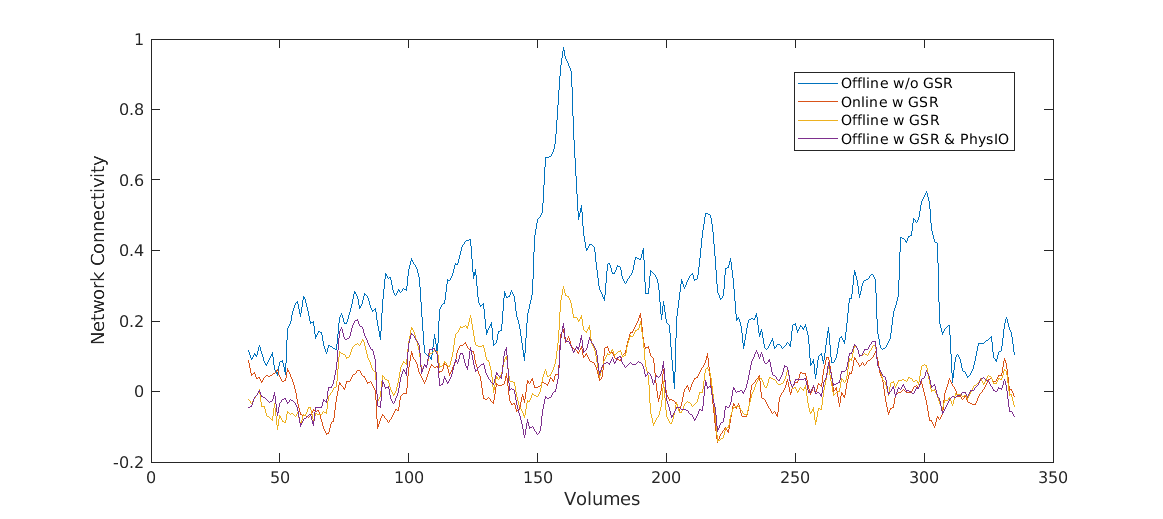


**Supplementary Figure 4**. Exemplary target network connectivity time course of a single run in a single participant with the different applied processing strategies. The online time course (red) was estimated during training while the other three were estimated with the offline processing pipeline. The strong influence of GSR on the network connectivity estimate is clearly visible. The differences between online and offline analyses and the additional effect of the PhysIO toolbox appear small in comparison.
